# Ultrasound-Guided Pigtail Catheter Drainage of Pleural Effusion in the Emergency Department: Effectiveness, Safety, and Clinical Implications

**DOI:** 10.3390/jcm14165704

**Published:** 2025-08-12

**Authors:** Aleksandra Szymczyk, Dominik Płaza, Mariusz Siemiński

**Affiliations:** 1 Department of Emergency Medicine, Medical University of Gdansk, 80-214 Gdansk, Poland; azszymczyk@gumed.edu.pl; 2 Emergency Department, University Clinical Centre, 80-214 Gdansk, Poland; dplaza@uck.gda.pl

**Keywords:** pleural effusion, pigtail catheter, emergency medicine

## Abstract

**Background**: Pleural effusion is a common clinical condition encountered in emergency departments and often requires timely therapeutic intervention. This study aimed to assess the effectiveness and safety of ultrasound-guided pigtail catheter drainage in patients presenting with symptomatic pleural effusion. **Methods:** We conducted a retrospective analysis of 134 drainage procedures performed in a tertiary hospital emergency department in 2024. Adult patients who underwent ultrasound-guided drainage were included regardless of primary diagnosis. **Results:** Clinical improvement was observed in 86.6% of patients, while radiological improvement—assessed only in cases with complete follow-up imaging—was seen in 56.0%. Procedure-related complications were rare (3.7%), and 50% of patients were discharged directly from the emergency department, highlighting the feasibility of ambulatory management. Nearly half of the patients had underlying malignancy and were receiving palliative care. **Conclusions:** While indwelling pleural catheters (IPCs) are typically used in long-term outpatient settings, our study focused on temporary pigtail catheter drainage performed in-hospital as a symptom-relieving intervention. The findings align with previous studies supporting the safety and effectiveness of small-bore catheter use in this context. Ultrasound-guided pigtail drainage represents a low-risk, patient-centered approach that can reduce the burden on inpatient services and enhance quality of care for individuals with advanced disease. This method may be considered a first-line option in selected patients presenting with large or symptomatic pleural effusions in acute care settings

## 1. Introduction

Pleural effusion refers to the pathological accumulation of fluid in the pleural space, resulting from a wide range of underlying conditions, including heart failure, malignancies, infections, liver cirrhosis, and autoimmune diseases. It is one of the most frequently encountered thoracic abnormalities in clinical practice and represents a significant diagnostic and therapeutic challenge, especially in emergency settings. Effusions can be classified as transudative or exudative, and accurate differentiation between them is essential for guiding appropriate treatment [1,2,3]. The mechanisms of pleural effusion formation involve an imbalance between fluid production and resorption. Transudates typically result from increased hydrostatic pressure or decreased oncotic pressure, as seen in congestive heart failure or liver cirrhosis. Exudative effusions, on the other hand, are commonly due to increased capillary permeability or direct pleural injury, as in malignancy, pneumonia, or pulmonary embolism [1,4,5]. Pleural effusion is a frequent clinical condition, with an estimated annual incidence of 1.5 million cases in the United States [6] and approximately 400,000 to 500,000 cases in Germany alone, reflecting its high burden across developed healthcare systems [1,7]. In the emergency department (ED), pleural effusion may be the first clinical manifestation of an underlying systemic or oncologic disease. It is also frequently encountered as a complication of acute cardiopulmonary conditions and requires rapid assessment and intervention [8,9]. Management of pleural effusion depends on its cause and the severity of symptoms. For many patients, conservative treatment focused on the underlying disease is sufficient. However, in symptomatic cases with large effusions causing dyspnea, therapeutic drainage is required. Increasingly, ultrasound-guided, percutaneous pigtail catheters are being used as a less invasive alternative to traditional large-bore chest tubes. These small-caliber drains offer advantages such as lower complication rates, greater patient comfort, and suitability for both inpatient and outpatient care [10,11,12,13,14]. Recent meta-analyses and clinical observations further reinforce the role of pigtail catheters across diverse patient populations. In adult trauma settings, pigtail catheters were found to result in higher initial drainage output and a significantly lower risk of requiring surgical intervention compared to traditional chest tubes, while maintaining similar failure rates [15]. In the context of burn injuries, where anterior thoracic access is often compromised, pigtail catheters have also proven effective as a salvage therapy when large-bore chest tubes failed [16]. Moreover, in patients with cardiac, renal, or hepatic dysfunction, small-caliber drainage methods have become essential due to their reduced procedural risk and compatibility with fragile pleural anatomy [7]. The emergency department plays a critical role in the initial diagnosis, stabilization, and management of patients with pleural effusion. Bedside ultrasound, combined with clinical evaluation, facilitates timely decision-making regarding observation, thoracentesis, or catheter drainage. Prompt intervention in cases of respiratory distress or hemodynamic instability is crucial for patient outcomes [4,9]. The aim of this study is to assess the effectiveness and safety of pleural effusion drainage performed in the emergency department using pigtail catheters.

## 2. Materials and Methods

This study is a retrospective observational analysis conducted in the clinical emergency department (ED) of a tertiary university hospital. The aim was to evaluate the safety and efficacy of ultrasound-guided pigtail catheter drainage in patients presenting with symptomatic pleural effusion. The analysis included all adult patients undergoing pleural drainage procedures between January and December 2024. Data were retrieved from the hospital’s electronic medical record system (CliniNet) and cross-referenced with procedural logs. Initially, all cases coded as “pleural fluid reservoir drainage under ultrasound guidance” were identified. Exclusion criteria included non-pleural drainages (e.g., abdominal or soft tissue procedures), erroneously entered orders, and cases disqualified by the interventional radiologist prior to drainage. Out of 176 drainage procedures reviewed, 134 were confirmed as pleural drainages meeting the inclusion criteria and were analyzed in detail. All procedures were performed by interventional radiologists using real-time ultrasound guidance and percutaneous pigtail catheters (typically 8–10 Fr), placed under local anesthesia with a sterile technique and the Seldinger method. Interventional radiologists were available 24 h a day, either on site during scheduled shifts or through an on-call system, which ensured timely drainage procedures in eligible patients. For large-volume effusions, staged evacuation was applied to reduce the risk of re-expansion pulmonary edema. Demographic and clinical data were extracted from procedural and discharge documentation. Variables included age, sex, chief complaint, respiratory symptoms, oxygen saturation, inflammatory markers (CRP, WBC), oncologic status (including whether the patient was receiving palliative care or chemotherapy), and pleural effusion characteristics (laterality, size, and presumed etiology). Post-procedure parameters included final oxygen saturation, need for continued oxygen therapy, subjective symptom relief, and discharge destination (home vs. inpatient admission). Subjective clinical improvement was defined as documented reduction in dyspnea or chest discomfort as reported in the physician’s post-procedure note. Symptom relief was assessed through physician evaluation based on a follow-up interview with the patient and bedside clinical observation. Indicators included subjective improvement in breathing, observed respiratory effort, and changes in oxygen saturation. A standardized symptom scoring system was not applied. All data were extracted retrospectively from narrative entries in the ED documentation. Radiological improvement was based on reduced fluid volume on follow-up chest imaging (ultrasound or chest X-ray). Clinical and safety outcomes were predefined based on symptom resolution, radiologic findings, discharge disposition, and post-procedure complications within 14 days. Descriptive statistics were used to summarize results. Continuous variables were reported as means or medians with standard deviations or interquartile ranges, and categorical variables were reported as counts and percentages. The study protocol was approved by the local Bioethics Committee. Given the retrospective nature of this study and the use of anonymized data, the requirement for individual informed consent was waived.

## 3. Results

In 2024, a total of 41,417 patients were admitted to the emergency department (ED), among whom 134 ultrasound-guided pigtail catheter drainages were performed for symptomatic pleural effusion. All procedures met the inclusion criteria and were included in the final analysis. The average age of the study population was 69.2 years (SD: 12.6), with a median age of 72 years and a range from 29 to 95. The 25th and 75th percentiles were 62 and 76 years, indicating that the majority of procedures were performed in elderly patients. Women accounted for 58% of the cohort. Malignancy was present in 52% of patients, and nearly half (47%) were referred from or currently under palliative care, highlighting the advanced clinical status of this population. Most effusions were unilateral (73.9%), while 27.6% were bilateral. Bilateral pleural fluid accumulation was more commonly observed in patients with suspected systemic diseases, such as congestive heart failure or late-stage malignancy. A concurrent diagnosis of pneumonia was confirmed in 23 patients (17.2%), which may have contributed to acute decompensation and prompted emergent drainage. On admission, oxygen therapy was required in 62 patients (46.3%), underscoring the severity of dyspnea and respiratory compromise. Clinical improvement, as documented by the treating physician and based on patient-reported symptom relief, was observed in 116 cases (86.6%). Subjective improvement was consistent across both oncologic and non-oncologic subgroups. In contrast, 18 patients (13.4%) showed no symptomatic change following drainage. Radiological improvement, assessed in patients with complete pre- and post-drainage imaging, was confirmed in 75 cases (56.0%). However, 10 patients (7.4%) demonstrated no radiologic change despite undergoing the procedure. In 49 cases (36.6%), radiologic data were incomplete or unavailable, precluding reliable evaluation. Follow-up imaging after drainage was not standardized and varied depending on the attending physician’s clinical judgment. In many cases, bedside ultrasound was performed directly after the procedure; in others, lack of further drainage and clinical improvement were deemed sufficient. Chest X-ray or repeat ultrasound was occasionally used for further evaluation. These pragmatic decisions reflect the operational reality of emergency care, where protocols may vary due to time constraints and patient throughput. A total of 67 patients (50%) were discharged directly from the ED following successful drainage and symptomatic stabilization, demonstrating the feasibility of ambulatory management. The remaining 66 patients (49.2%) required hospital admission due to comorbidities, persistent symptoms, or social indications. Procedure-related complications were rare and occurred in only five patients (3.7%). These included minor bleeding and catheter dislodgement, and no major events such as tension pneumothorax or hemothorax were observed. Within 14 days of the initial procedure, 15 patients (11.2%) returned to the ED, most often due to symptom recurrence or catheter-related issues. Re-drainage was required in a minority of cases, and no emergency surgical interventions were documented. Clinical efficacy was evaluated using three predefined outcome measures: symptom relief, radiological improvement, and avoidance of hospital admission. Safety was assessed by monitoring for procedure-related complications and revisits.

## 4. Discussion

The results of this retrospective study confirm the high efficacy and safety of ultrasound-guided pigtail catheter drainage in the management of symptomatic pleural effusions in the emergency department (ED) setting. The observed clinical improvement rate of 86.6% and radiological improvement in 61.2% of cases are consistent with previous reports, where clinical success rates for small-bore catheter drainage ranged between 80 and 90%, depending on the underlying cause of effusion [3,11]. Our findings are aligned with those of Benton et al., who compared small-bore (6–12 F) and large-bore (20–32 F) chest tubes in the treatment of spontaneous pneumothorax. While the clinical resolution rates were comparable (80–88%), the complication rate was significantly higher in the large-bore group (32% vs. 21%), with infections being the most common issue [17]. Similarly, Hassani et al. demonstrated that 81% of patients managed with 8 F pigtail catheters and Heimlich valves for primary spontaneous pneumothorax were successfully discharged directly from the ED, further supporting the feasibility of ambulatory management [18]. In our study, 50% of patients were also discharged directly from the ED, echoing findings from Rivera et al., who showed that pigtail catheters were associated with lower complication rates—particularly fibrothorax—while maintaining similar efficacy to that of traditional chest tubes [19]. Moreover, Kulvatunyou et al. found no significant differences in ventilation duration, ICU stay, or number of drainage days between the two methods in trauma patients [20]. The low complication rate observed in our cohort (3.7%) aligns with global data on the safety of small-bore catheter use. Munavvar et al. concluded that both indwelling pleural catheters (IPCs) and traditional chest tubes are generally safe; however, pigtail catheters offer additional advantages in cancer patients, including ease of use, patient comfort, and potential for outpatient care [21]. While the AABIP guidelines highlight the value of indwelling pleural catheters (IPCs) in the long-term management of malignant pleural effusion, particularly in palliative care, our study focused exclusively on the use of short-term pigtail catheters in the emergency setting. These are distinct approaches with different clinical purposes [22]. A recent study by Türk et al. also confirmed the safety and feasibility of tunneled pigtail catheters in patients with refractory pleural effusion, showing 100% technical success and an acceptable complication rate in both malignant and benign cases [23]. In our cohort, 52% of patients had underlying malignancy, and 47% were under palliative care at the time of the procedure. These numbers highlight the critical importance of low-risk, symptom-relieving interventions that avoid prolonged hospitalization. As emphasized by recent guidelines, including those from the British Thoracic Society and Munavvar et al., clinical decision-making in malignant pleural effusion (MPE) must be patient-centered, considering performance status, lung re-expansion potential, and symptom burden [9,21]. Furthermore, as Scott and Phil point out, the median survival for patients with MPE ranges from 3 to 12 months, making symptom control and quality of life essential therapeutic goals [24]. Our findings support the use of pigtail catheters as a front-line option in such cases, especially given their compatibility with home-based drainage protocols. It is also important to consider that pigtail catheter efficacy is influenced by the nature of the pleural fluid. Due to their smaller internal diameter, they are most effective in draining air or low-viscosity serous effusions. In cases of septated or thick empyema, larger bore drains or adjunctive therapies may be required [1]. Proper patient selection, guided by bedside ultrasound and lateral decubitus radiography, can mitigate these limitations. Our findings are further supported by recent analyses emphasizing the comparable or superior outcomes of pigtail catheters in various complex scenarios. For example, a 2023 meta-analysis of thoracic trauma patients demonstrated a significantly lower need for video-assisted thoracic surgery (VATS) among those treated with pigtail catheters compared to chest tubes, despite equivalent overall failure rates [15]. In burn victims, pigtail catheters were successfully used when traditional large-bore tubes failed, highlighting their utility even in anatomically and clinically challenging cases [16]. Furthermore, recent reviews underscore their increasing role in the management of nonmalignant pleural effusions related to organ failure, where conservative and symptom-focused management is essential [7]. These findings, consistently with the previous literature, support the use of pigtail catheter drainage as a safe and practical option for the acute management of pleural effusion in the emergency setting—particularly in frail or palliative patients, where minimally invasive approaches are most beneficial. This study has several limitations that must be acknowledged. First, its retrospective design introduces potential biases related to incomplete documentation, case selection, and underreporting of complications or negative outcomes. Radiologic follow-up was not available in all cases, which may have led to overestimation of success rates. Second, the analysis was conducted in a single academic center with access to on-site interventional radiologists and ultrasound guidance, which may limit generalizability to smaller hospitals or resource-limited settings. Third, although the study population reflects real-world ED practice—including a high proportion of patients with advanced malignancy and organ failure—it remains heterogeneous and may not permit direct comparisons between clinical subgroups. Fourth, the absence of a comparator group treated with large-bore chest tubes prevents direct evaluation of relative efficacy. Fifth, although half of the patients were discharged directly from the ED, this approach was only feasible in selected cases. Patients who were unable to manage the catheter safely at home due to clinical, cognitive, or social reasons were admitted to the hospital. This limits the generalizability of outpatient applicability. Finally, the assessment of symptom relief was based on physician documentation and not on standardized patient-reported outcome measures, which limits the precision of subjective outcome evaluation. Despite these limitations, this study offers valuable insight into the practical use of pigtail catheters in the ED setting and supports their role as a safe, effective, and patient-centered intervention for pleural effusion management.

## 5. Conclusions

Effective management of symptomatic pleural effusion requires timely and appropriate intervention, particularly in the emergency department setting, where clinical urgency and resource constraints often shape therapeutic decisions. In recent years, minimally invasive techniques—such as ultrasound-guided pigtail catheter drainage—have gained increasing recognition for their favorable safety profile and clinical efficacy. The growing role of small-bore catheter drainage reflects a broader shift in modern medicine: a move away from high-burden interventions toward patient-centered approaches tailored to clinical condition, therapeutic goals, and overall prognosis. For patients with advanced illness or malignancy, rapid relief of dyspnea using short-term drainage—such as pigtail catheters in emergency settings—offers an important bridge in care. However, long-term management of malignant pleural effusions may still require dedicated approaches such as indwelling pleural catheters. Expanding the use of point-of-care ultrasound, enhancing procedural competencies among emergency physicians, and integrating acute care with quality-of-life-focused decision-making are essential directions for the evolution of emergency medicine. In this context, pigtail catheter drainage is not only a technically effective intervention but also a practical, compassionate, and system-responsive solution to the complex challenges of modern healthcare.

## Data Availability

Data are available from the corresponding author on individual request.

## References

[1] Light R.W. (1992). Pleural diseases. Dis.-a-Mon..

[2] Porcel J.M. (2011). Pearls and myths in pleural fluid analysis. Respirology.

[3] Roberts J.S., Bratton S.L., Brogan T.V. (1998). Efficacy and complications of percutaneous pigtail catheters for thoracostomy in pediatric patients. Chest.

[4] Ferreiro L., Toubes M.E., San José M.E., Suárez-Antelo J., Golpe A., Valdés L. (2020). Advances in pleural effusion diagnostics. Expert. Rev. Respir. Med..

[5] Sahn S.A. (1982). The Differential Diagnosis of Pleural Effusions. West. J. Med..

[6] Feller-Kopman D., Light R. (2018). Pleural Disease. N. Engl. J. Med..

[7] Jany B., Welte T. (2019). Pleural Effusion in Adults-Etiology, Diagnosis, and Treatment. Dtsch. Arztebl. Int..

[8] Lee Y.C.G., Davies R.J.O. (2016). Management of Pleural Effusions in Acute Medical Settings: A practical guide. CPD. J. Acute. Med..

[9] Roberts M.E., Rahman N.M., Maskell N.A., Bibby A.C., Blyth K.G., Corcoran J.P., Edey A., Evison M., De Fonseka D., Hallifax R. (2023). British Thoracic Society Guideline for pleural disease. Thorax.

[10] Jayakrishnan B., Kashoob M., Al-Sukaiti R., Al-Mubaihsi S., Kakaria A., Al-Ghafri A., Al-Lawati Y. (2021). Percutaneous Ultrasound-guided Pigtail Catheter for Pleural Effusions: Efficacy and Safety. Oman. Med. J..

[11] Khare R., anand K., Agrawal P., Yadav A. (2023). Comparative analysis of pigtail catheter versus intercostal tube drainage for pleural effusion: A tertiary centre study. Int. Surg. J..

[12] Mehra S., Heraganahally S., Sajkov D., Morton S., Bowden J. (2020). The effectiveness of small-bore intercostal catheters versus large-bore chest tubes in the management of pleural disease with the systematic review of literature. Lung. India..

[13] Sartori S., Tombesi P., Tassinari D., Ceccotti P., Nielsen I., Trevisani L., Abbasciano V. (2004). Sonograpically guided small-bore chest tubes and sonographic monitoring for rapid sclerotherapy of recurrent malignant pleural effusions. J. Ultrasound Med..

[14] Wei Y.H., Lee C.H., Cheng H.N., Tsao L.T., Hsiao C.C. (2014). Pigtail catheters versus traditional chest tubes for pneumothoraces in premature infants treated in a neonatal intensive care unit. Pediatr. Neonatol..

[15] Harding W.C., Halawa A.R., Aiche M.M., Zafar B., Ali H.J.R., Bashoura L., Faiz S.A. (2025). Pleural Effusion: Shedding Light on Pleural Disease Beyond Infection and Malignancy. Medicina.

[16] Sebastian R., Ghanem O., Diroma F., Milner S.M., Gerold K.B., Price L.A. (2015). Percutaneous pigtail catheter in the treatment of pneumothorax in major burns: The best alternative? Case report and review of literature. Burns.

[17] Benton I.J., Benfield G.F.A. (2009). Comparison of a large and small-calibre tube drain for managing spontaneous pneumothoraces. Respir. Med..

[18] Hassani B., Foote J., Borgundvaag B. (2009). Outpatient management of primary spontaneous pneumothorax in the emergency department of a community hospital using a small-bore catheter and a heimlich valve. Acad. Emerg. Med..

[19] Rivera L., O’Reilly E.B., Sise M.J., Norton V.C., Sise C.B., Sack D.I., Swanson S.M., Iman R.B., Paci G.M., Antevil J.L. (2009). Small catheter tube thoracostomy: Effective in managing chest trauma in stable patients. J. Trauma Acute Care Surg..

[20] Kulvatunyou N., Erickson L., Vijayasekaran A., Gries L., Joseph B., Friese R.F., O’Keeffe T., Tang A.L., Wynne J.L., Rhee P. (2014). Randomized clinical trial of pigtail catheter versus chest tube in injured patients with uncomplicated traumatic pneumothorax. Br. J. Surg..

[21] Munavvar M., Bodtger U., Carus A., Cordovilla R., Naik S., Salud A., Porcel J.M. (2024). Current Trends in Treating Malignant Pleural Effusion: Evidence, Guidelines, and Best Practice Recommendations. JCO Oncol. Pract..

[22] Miller R.J., Chrissian A.A., Lee Y.C.G., Rahman N.M., Wahidi M.M., Tremblay A., Hsia D.W., Almeida F.A., Shojaee S., Mudambi L. (2020). AABIP Evidence-informed Guidelines and Expert Panel Report for the Management of Indwelling Pleural Catheters. J. Bronchol. Interv. Pulmonol..

[23] Türk Y., Devecioğlu İ., Yıldızhan İ., Arslan B.C., Arıbaş B.K. (2022). Tunneled Uncuffed Pigtail Drainage Catheter Placement in Patients with Refractory Ascites or Pleural Effusion: A Single-Center Experience. Cardiovasc. Interv. Radiol..

[24] Scott H., Phil R. (2024). What You Need to Know About: The Management of Malignant Pleural Effusion. Br. J. Hosp. Med..

